# Comparison of the overall survival of proximal and distal gastric cancer after gastrectomy: a systematic review and meta-analysis

**DOI:** 10.1186/s12957-021-02126-4

**Published:** 2021-01-19

**Authors:** Jiaming Xue, Huiliang Yang, Shanshan Huang, Tingting Zhou, Xiangwen Zhang, Guo Zu

**Affiliations:** 1 Department of Gastroenterology Surgery, The Dalian Municipal Central Hospital Affiliated of Dalian Medical University, No. 826 Southwest Road Shahekou District, Dalian, 116033 P.R. China; 2 Dalian Medical University, Dalian, 116011 P.R. China; 3 Department of Neurology, The First Affiliated Hospital of Dalian Medical University, Dalian, 116011 P.R. China

**Keywords:** Gastric cancer, Proximal gastric cancer, Distal gastric cancer, Prognosis, Meta-analysis

## Abstract

**Background:**

The aim of this study was to investigate the overall survival (OS) between proximal gastric cancer (PG) and distal gastric cancer (DG) patients after gastrectomy.

**Methods:**

Articles on the prognostic study of PG and DG patients after gastrectomy were collected from the PubMed, EMBASE, Web of Science, Cochrane Library, China National Knowledge Infrastructure (CNKI), Wanfang, and VIP databases from the date of establishment until December 2020. The data were statistically analyzed by Stata software (version 11.0, StataCorp).

**Results:**

A total of 10 articles met the inclusion criteria. Meta-analysis showed that the 1-, 3- and 5-year OS rates of PG patients were significantly lower than those of DG patients (RR = 0.898, 95% CI: 0.825 to 0.977, *P* = 0.013; RR = 0.802, 95% CI: 0.708 to 0.909, *P* = 0.001; RR = 0.736, 95% CI: 0.642 to 0.844, *P* = 0.000). After subgroup analysis according to different countries, the combined RR values of were as follows: 1-year OS: eastern countries: RR = 0.966, 95% CI: 0.944 to 0.988, *P* = 0.003, western countries: RR = 0.687, 95% CI: 0.622 to 0.759, *P* = 0.000; 3-year OS: eastern countries: RR = 0.846, 95% CI: 0.771 to 0.929, *P* = 0.000, western countries: RR = 0.742, 95% CI: 0.399 to 1.382, *P* = 0.348; and 5-year OS: eastern countries: RR = 0.798, 95% CI: 0.716 to 0.889, *P* = 0.000, western countries: RR = 0.646, 95% CI: 0.414 to 1.008, *P* = 0.054.

**Conclusion:**

In terms of 1-, 3-, and 5-year OS, PG patients had lower rates than DG patients and the eastern countries/western countries subgroup, but there were no significant differences in 3- and 5-year OS for the western countries. These results merit further clinical validation in future studies.

(Registration ID: UMIN000040393; Date of registration: 2020/05/13)

## Introduction

Gastric cancer (GC) is one of the most common cancers of the digestive system [1]. A total of 1,000,000 new GC cases and 783,000 GC-related deaths occurred worldwide in 2018 [2]. Among them, the incidence rates in East Asian countries such as Korea, Japan, Mongolia, and China are the highest [3], affecting between 40 and 60 per 100,000 inhabitants [4]. This difference may be caused by *Helicobacter pylori* infection, poor diet, and unhealthy habits, such as smoking or alcohol consumption [5].

Common types of GC include proximal (PG) and distal GC (DG). PG is defined as cancers with the center located in the cardia or fundus, whereas DG is defined as lesions in the body, antrum, or pylorus [6]. Some early GC patients cannot be easily diagnosed because the early symptoms are not obvious or for other reasons. At this time, surgery is still an important method to improve their survival rate of GC patients [7]. Depending on the location of the tumor, different surgical methods are usually selected, such as proximal gastrectomy or distal gastrectomy. There have been many reports about PG and DG, but their findings are quite different. For instance, Choi et al. and Fatih et al. reported that DG patients had higher 5-year overall survival (OS) rates than PG patients [8–10]. This may be related to the more insidious early symptoms in PG patients, and many PG patients are already at an advanced stage when they diagnosed, which leads to worse prognosis. However, Qin et al. and Laurence et al. reported no difference in OS between DG and PG [11, 12]. The different prognosis of DG and PG patients are of great significance in guiding surgical treatment. Therefore, to explore the differences in prognosis between PG and DG patients, we extracted and integrated the 1-, 3-, and 5-year OS rates from articles and performed a systematic review and meta-analysis, with the aim of providing evidence for the prognostic evaluation of GC after gastrectomy.

## Methods

This study is reported in line with the Preferred Reporting Items for Systematic Reviews and Meta-Analyses (PRISMA) [13] and Assessing the Methodological Quality of Systematic Reviews (AMSTAR) guidelines [14].

### Data sources and searches

We systematically searched the PubMed, EMBASE, Web of Science, Cochrane Library, China National Knowledge Infrastructure (CNKI), Wanfang, and VIP databases from the date of establishment until December 2020. The search terms were follows: “proximal gastric cancer” and “distal gastric cancer” and (“prognosis” or “overall survival” or OS) and (gastrectomy or surgery). In addition, we reviewed the references of all selected articles to identify other relevant studies.

### Inclusion and exclusion criteria

The inclusion criteria were as follows: (1) randomized controlled trials (RCTs)/cohort studies/observational studies, (2) comparison of prognosis between PG and DG patients, and (3) recorded at least one of the following effect sizes: 1-, 3-, or 5-year OS.

The exclusion criteria were as follows: (1) nongastric cancer; (2) failed to include both PG and DG patients; (3) too little data to use; (4) case reports, reviews, and comments; and (5) not following gastrectomy.

### Data extraction and quality assessment

Two reviewers independently searched all the preliminary inclusion reports for information extraction and evaluation. The extracted information included the following: first author, country, publication year, number of patients, and 1-, 3-, and 5-year OS. The Newcastle-Ottawa Scale (NOS) was used to evaluate the quality of the included articles. The evaluation contents were “selection,” “comparability” and “exposure/outcome”, and every high-quality component given a “star”. The differences were settled by discussion with all the authors together.

### Statistical analysis

All data were analyzed with Stata software (version 11.0, StataCorp). The relative risk (RR) with a 95% confidence interval (CI) was used to analyze the survival rate. Heterogeneity was evaluated by the Q value and *I*^*2*^ test. The corresponding combination method according to the results of the heterogeneity test: if there was no significant heterogeneity among groups (*P* ≥ 0.1), the fixed effect model was selected for combination and analysis; if there was significant heterogeneity among groups (*P* < 0.1), the random effect model was selected for combination and analysis. The source of heterogeneity among studies was explored by subgroup and sensitivity analyses. Finally, publication bias was assessed by Begg’s test and Egger’s test. A level of *α* = 0.05 was considered significant.

## Results

### Eligible studies

**Fig. 1:**
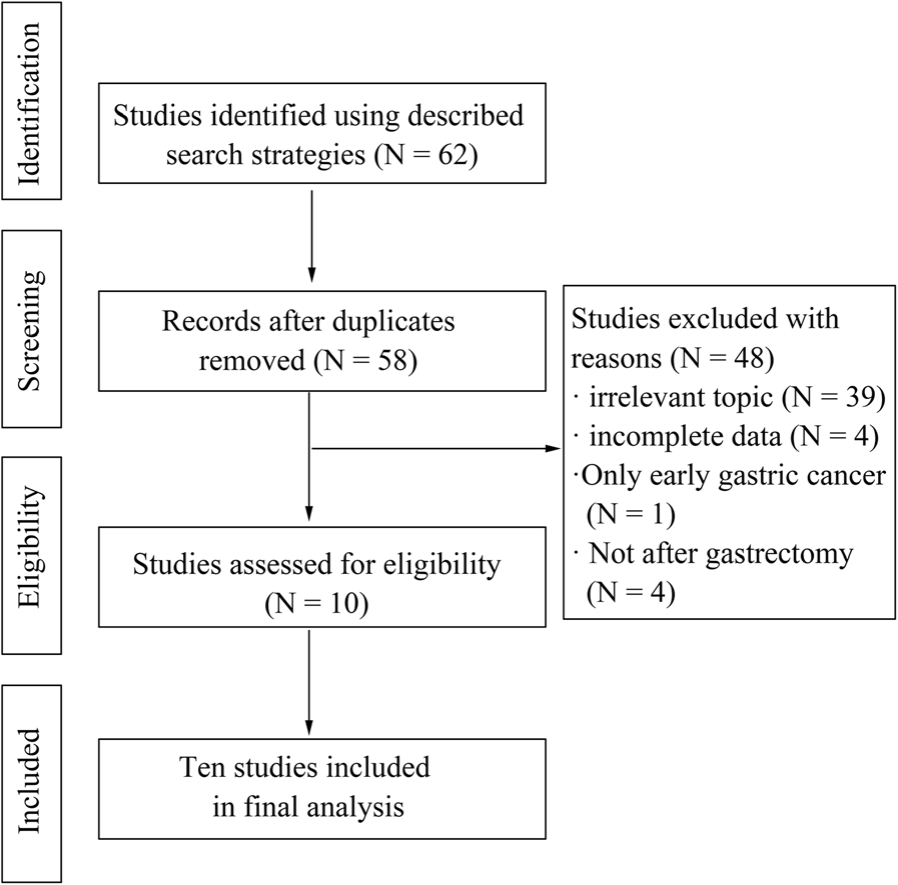
Flow chart of the identification process for eligible studies

We initially retrieved 62 articles, and then excluded 4 duplicate articles. By reading the titles and abstracts, 39 irrelevant topic articles were excluded. Then, by reading the full texts of the remaining articles, according to the inclusion and exclusion criteria, we excluded 4 articles with incomplete data, 1 article with only early cases, and 4 articles without gastrectomy. Finally, we included 10 studies, with 3 in Chinese and 7 in English [8, 10–12, 15–20]. Figure 1 shows the flow chart of the selection process. Table 1 records the data from each study. We evaluated the studies by NOS, and the NOS score obtained a range of 5–6 stars, indicating that the quality of the included studies was at a medium to high level (Table 2).

**Table 1:** Summary of data for each study

Author	Published year	Country	PG							DG						
			Total	1 year		3 year		5 year		Total	1 year		3 year		5 year	
				Survival	Death	Survival	Death	Survival	Death		Survival	Death	Survival	Death	Survival	Death
T eruyuki Sakaguchi	1998	Japan	141	119	22	91	50	83	58	250	224	26	192	58	174	76
Jang Kyu Choi	2015	Korea	48	46	2	38	10	37	11	271	267	4	257	14	245	26
Qin Huang	2015	China	83	81	2	74	9	65	18	165	157	8	154	11	139	26
Jun Chul Park	2010	Korea	503	463	40	373	130	329	174	2690	2555	135	2271	419	2122	568
Laurence Bedin da COSTA	2016	Brazil	69	37	32	29	40	24	45	209	125	17	85	124	67	142
Fabio Pacelli	2001	Italy	187					33	154	520					189	331
Pompiliu Piso	2000	Germany	250	154	96	85	165	63	187	282	243	39	175	107	133	149
Chong Li	2005	China	25	17	8	9	16	6	19	84	63	21	41	43	35	49
Wenhua Zhan	2002	China	133	112	21	55	78	38	95	232	208	24	152	80	111	121
Jianshan Hong	2005	China	152					42	110	258					122	136

**Table 2:** NOS score sheet

	Selection	Comparability	Outcome
T eruyuki Sakaguchi 1998	★★★☆	☆☆	★★☆
Jang Kyu Choi 2015	★★★☆	☆☆	★★☆
Qin Huang 2015	★★★☆	☆☆	★★★
Jun Chul Park 2010	★★★☆	☆☆	★★☆
Laurence Bedin da COSTA 2016	★★★☆	☆☆	★★★
Fabio Pacelli 2001	★★★☆	☆☆	★★☆
Pompiliu Piso 2000	★★★☆	☆☆	★★☆
Chong Li 2005	★★★☆	★☆	★★☆
Wenhua Zhan 2002	★★★☆	☆☆	★★★
Jianshan Hong 2005	★★★☆	☆☆	★★☆

### Outcomes of the meta-analysis

**Fig. 2:**
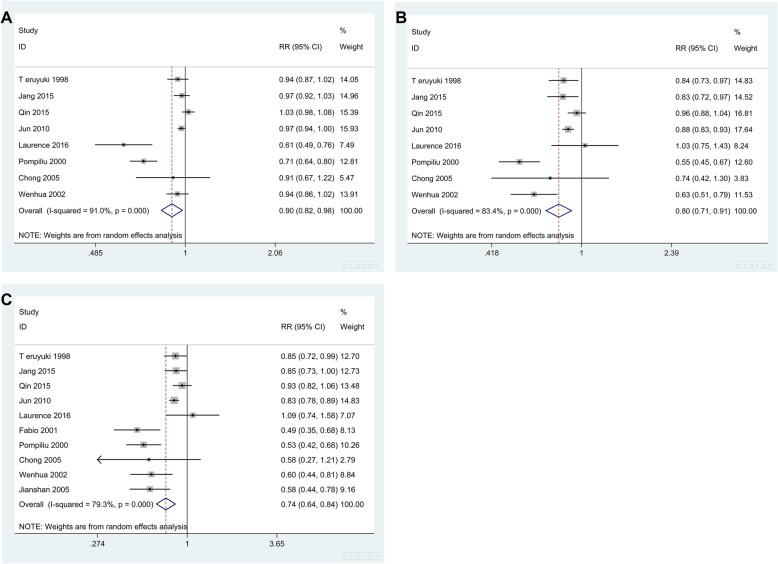
Forest graph of OS between PG and DG patients. **a** 1-year OS. **b** 3-year OS. **c** 5-year OS

The 1-year OS was described in 8 studies [8, 11, 12, 15–19]. The results suggested that there was significant heterogeneity (*Q* = 77.61, *I*^*2*^ *=* 91.0%, *P* = 0.000) among the groups in the fixed effect model. Therefore, we used the random effect model to analyze the data. The results were RR = 0.898, 95% CI: 0.825 to 0.977. The test result was statistically significant (*P* = 0.013) (Table 3 and Fig. 2a) and suggested a difference in the 1-year OS between PG and DG patients, namely, the 1-year survival rate of PG patients was lower than that of DG patients.

**Table 3:** RR value of OS obtained by random model

Study (year)	1 year		3 year		5 year	
	RR (95% CI)	Weight (%)	RR (95% CI)	Weight (%)	RR (95% CI)	Weight (%)
T eruyuki Sakaguchi (1998)	0.942 (0.867–1.023)	14.05	0.840 (0.731–0.967)	14.83	0.846 (0.720–0.993)	12.70
Jang Kyu Choi (2015)	0.973 (0.915–1.034)	14.96	0.835 (0.720–0.968)	14.52	0.853 (0.727–1.000)	12.73
Qin Huang (2015)	1.026 (0.977–1.076)	15.39	0.955 (0.877–1.040)	16.81	0.930 (0.815–1.060)	13.48
Jun Chul Park (2010)	0.969 (0.943–0.996)	15.93	0.878 (0.832–0.927)	17.64	0.829 (0.776–0.886)	14.83
Laurence Bedin (2016)	0.609 (0.485–0.765)	7.49	1.033 (0.749–1.426)	8.24	1.085 (0.743–1.584)	7.07
Pompiliu Piso (2000)	0.715 (0.641–0.797)	12.81	0.548 (0.451–0.666)	12.60	0.534 (0.417–0.684)	10.26
Chong Li (2005)	0.907 (0.674–1.219)	5.47	0.738 (0.418–1.300)	3.83	0.576 (0.274–1.210)	2.79
Wenhua Zhan (2002)	0.939 (0.862–1.023)	13.91	0.631 (0.505–0.789)	11.53	0.597 (0.442–0.806)	8.84
Fabio Pacelli (2001)	–	–	–	–	0.486 (0.349–0.675)	8.13
Jianshan Hong (2005)	–	–	–	–	0.584 (0.438–0.779)	9.16
Overall	0.898 (0.825–0.977)	100.00	0.802 (0.708–0.909)	100.00	0.736 (0.642–0.844)	100.00

Heterogeneity: 1-year survival: *Q* = 77.61, *I*^*2*^ *=* 91.0%, *P* = 0.000. 3-year survival: *Q* = 42.13, *I*^*2*^ *=* 83.4%, *P* = 0.000. 5-year survival: *Q* = 43.41, *I*^*2*^ *=* 79.3%, *P* = 0.000. *z* test: 1-year survival: *z* = 2.49, *P* = 0.013. 3-year survival: *z* = 3.46, *P* = 0.001. 5-year survival: *z* = 4.39, *P* = 0.000

The 3-year OS was also described in 8 studies [8, 11, 12, 15–19]. The results of the random effect model analysis showed that RR = 0.802, 95% CI: 0.708 to 0.909, and the results were statistically significant (*P* = 0.001) (Table 3 and Fig. 2b). These findings suggested that the 3-year survival rate of PG patients was lower than that of DG patients.

The 5-year survival rate was described in 10 studies [8, 10–12, 15–20]. The results of the random effect model analysis showed that RR = 0.736, 95% CI: 0.642 to 0.844, *P* = 0.000 (Table 3 and Fig. 2c). Thus, PG patients had a worse prognosis in terms of the 5-year OS.

### Subgroup analysis

**Fig. 3:**
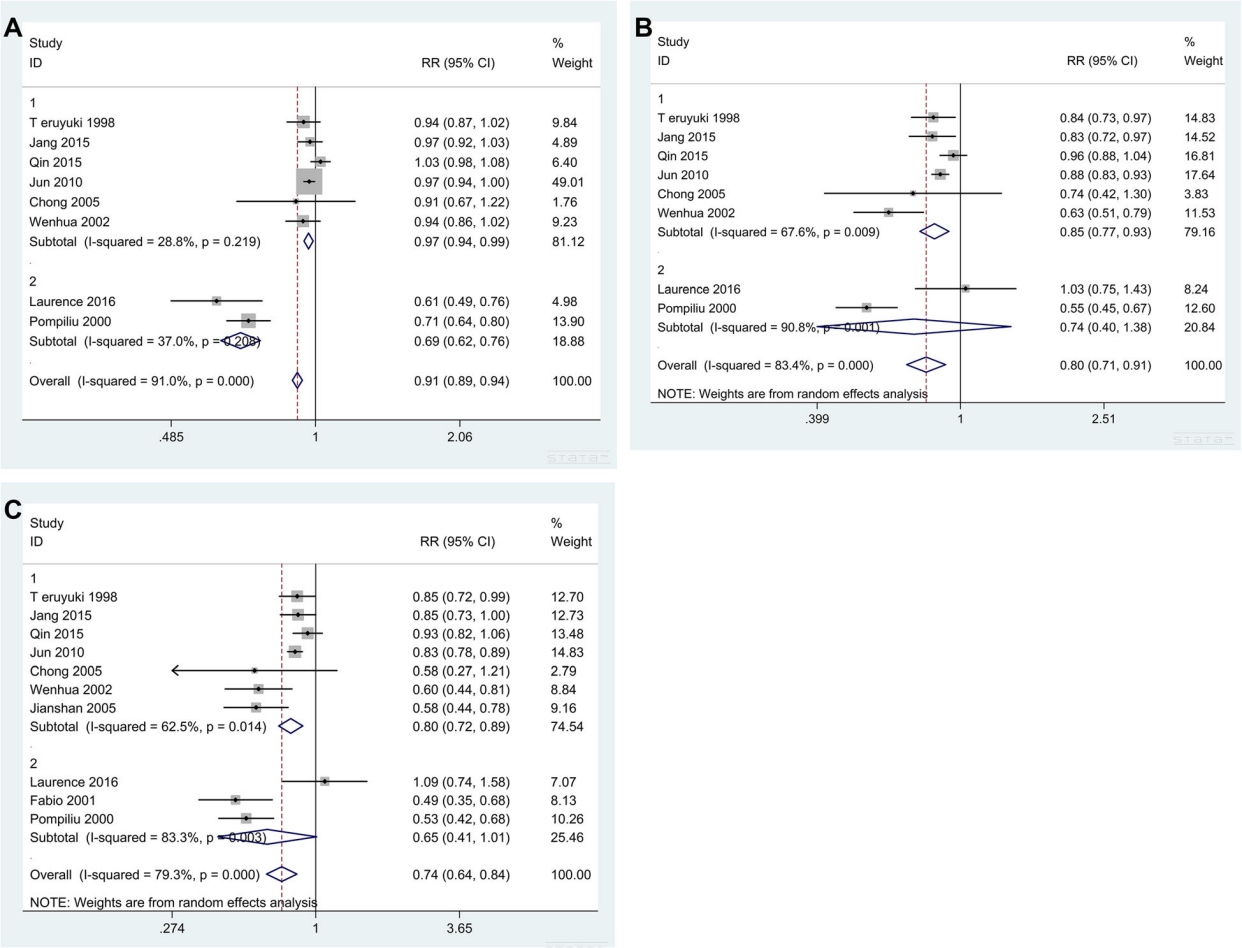
Results of subgroup analysis of 1-year (**a**), 3-year (**b**), and 5-year OS (**c**) (group 1: eastern countries; group 2: western countries)

The 1-year OS was described in 8 studies. Due to the high degree of heterogeneity, we conducted subgroup analysis to explore possible sources. We divided the studies into two subgroups according to their countries: group 1 (eastern countries) and group 2 (western countries). Figure 3a shows the results of the subgroup analysis of 1-year OS. The heterogeneity testing in each subgroup revealed that there was no heterogeneity among the studies (1. *Q* = 7.02, *I*^*2*^ *=* 28.8%, *P* = 0.219; 2. *Q* = 1.59, *I*^*2*^ *=* 37%, *P* = 0.208). Therefore, we used a fixed effect model to merge RR (1. RR = 0.966, 95% CI: 0.944 to 0.988, *P* = 0.003; 2. RR = 0.687, 95% CI: 0.622 to 0.759, *P* = 0.000), which suggested that the 1-year OS of PG patients was lower than that of DG patients.

In terms of the 3-year OS, the results of subgroup analysis suggested that there was still heterogeneity among the studies (1. *Q* = 15.44, *I*^*2*^ *=* 67.6%, *P* = 0.009; 2. *Q* = 10.91, *I*^*2*^ *=* 90.8%, *P* = 0.001). The RR values combined with the random effect model were (1) RR = 0.846, 95% CI: 0.771 to 0.929, *P* = 0.000, and (2) RR = 0.742, 95% CI: 0.399 to 1.382, *P* = 0.348 (Fig. 3b). Thus, the 3-year OS of PG patients was lower than that of DG patients in eastern countries, but there was no statistical significance in western countries.

When analyzing the 5-year OS of patients, the results of subgroup analysis suggested that there was still heterogeneity among the studies (1. *Q* = 16.02, *I*^*2*^ *=* 62.5%, *P* = 0.014; 2. *Q* = 11.95, *I*^*2*^ *=* 83.3%, *P* = 0.003). The combined RR values were (1) RR = 0.798, 95% CI: 0.716 to 0.889, *P* = 0.000, and (2) RR = 0.646, 95% CI: 0.414 to 1.008, *P* = 0.054 (Fig. 3c). Thus, the 5-year OS of PG patients was lower than that of DG patients in eastern countries, but there was no statistical significance in western countries.

### Publication bias

**Fig. 4:**
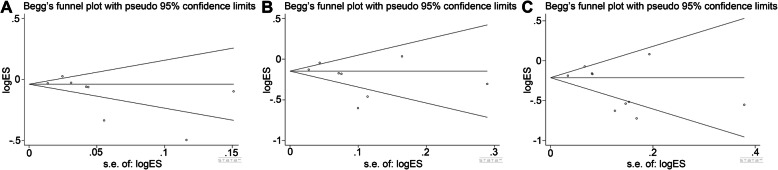
Begg’s test and Egger’s test of 1-year, 3-year, and 5-year OS. **a** 1-year OS. **b** 3-year OS. **c** 5-year OS

Publication bias was analyzed by Begg’s test and Egger’s test in our study. The results of Begg’s test and Egger’s test of 1-year OS (Begg’s test: *z* = 1.86, *P* = 0.063; Egger’s test: *P* = 0.106), 3-year OS (Begg’s test: *z* = 1.11, *P* = 0.266; Egger’s test: *P* = 0.231), and 5-year OS (Begg’s test: *z* = 1.07, *P* = 0.283; Egger’s test: *P* = 0.133) suggested that there was no significant publication bias (Fig. 4).

### Sensitivity analysis

**Fig. 5:**
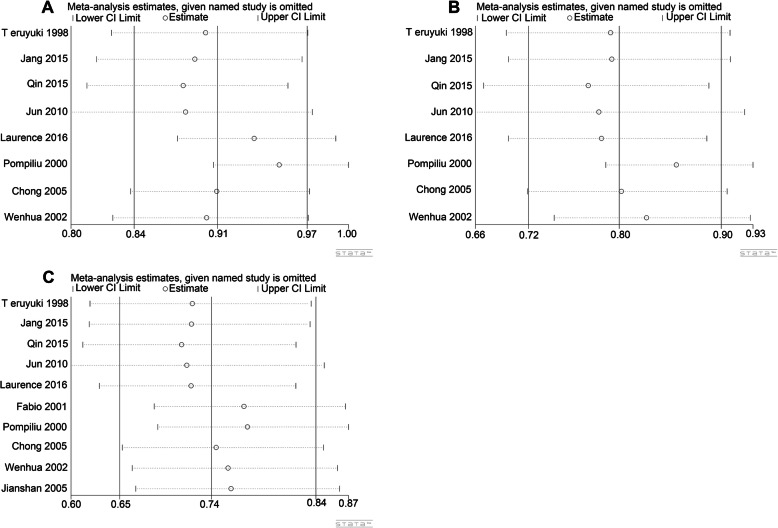
Sensitivity analysis of the overall pooled study. **a** 1-year OS. **b** 3-year OS. **c** 5-year OS

To explore the potential heterogeneity from any single included study, we carried out a sensitivity analysis, and the results are shown in Fig. 5. This sensitivity analysis indicated that the conclusions were not significantly affected by removing any single study.

## Discussion

GC is one of the most important cancers worldwide. It became the fifth most frequently diagnosed cancer and the third leading cause of cancer death worldwide in 2018 [2]. Although the incidence rate of GC has been reported to decline in some areas in recent years, GC is still one of the risk factors affecting global health, and the pathogenesis of GC is multifactorial [4, 21, 22]. With the rapid development of diagnosis and treatment of GC, surgery is still the major treatment for GC patients. PG and DG are the most common types of GC. Despite a decline in incidence of GC in western countries over the past decades, the incidence of PG is still increasing [23, 24]. This may be related to many factors such as *Helicobacter pylori* infection and eating habits [5, 25–28]. Many studies have reported the prognosis of PG and DG. It is expected that the prognosis of PG and DG will gradually increase with the increased availability of patient diagnostic facilities, improved effectiveness of multimodal treatment, promotion of cancer screening and early detection programs, and emerging surgical approaches. However, the results remain controversial. In our study, after meta-analysis and subgroup analysis, we found that the 1-year OS of PG patients was lower than that of DG patients, and the 3- and 5-year OS rates of PG patients were lower than those of DG patients in eastern countries, but there were no significant differences in western countries.

The reasons why PG patients have worse OS than DG patients have been investigated and are multifaceted. First, many PG patients do not have obvious or specific early symptoms, and detection is sometimes difficult with gastroscopy; even targeted biopsy may be less accurate, resulting in some patients not being diagnosed until the advanced stage of cancer [12]. PG patients with esophageal invasion (especially stage T2) have been reported to have a poor prognosis, which may be associated with a higher incidence of lymph node invasion, leading to a wider spread of the tumor [15, 29]. Similarly, Qin et al. suggested that patients with PG had more aggressive tumors, leading to poorer prognosis [17, 30, 31]. Furthermore, p53 gene mutation has been reported to be an independent prognostic factor for GC, and the survival rate of patients with p53-positive tumors is often lower than that of patients with p53-negative tumors. They detected p53 more frequently in PG patients, which may explain the poor prognosis of PG patients compared to DG patients [15, 32, 33]. In addition, due to their different anatomical locations, the complexity of surgery in PG patients is significantly greater than that in DG patients, which may also be one of the factors contributing to the difference in prognosis [5]. In summary, these factors may ultimately lead to worse prognosis in PG patients than in DG patients.

Moreover, it is meaningful to further identify the reason for the difference in OS between PG and DG to develop better early screening, diagnosis, and treatment strategies for GC patients. At the same time, comparing the differences in screening, diagnosis, treatment strategies, and surgical methods in GC between eastern and western countries may provide suggestions for improving the OS of PG in eastern countries. Our results suggest that there is no significant difference in the 3- and 5-year OS rates between PG and DG patients in western countries. This may be related to the differences in the diagnosis and treatment of GC between the East and the West, such as earlier general survey, the basis of pathological diagnosis, the operation mode, and the adjuvant treatment plan [34, 35], but further clinical validation in future studies will be needed to confirm these possibilities.

Although our study used strict inclusion and exclusion criteria for article screening, there are still limitations. First, the impact of different surgical methods and adjuvant treatment schemes on the OS of patients with PG and DG patients was not considered. Second, we did not find the source of heterogeneity through sensitivity analysis and subgroup analysis, but we could confirm that regional factors were not the source of heterogeneity in this result. Perhaps the heterogeneity was related to other causes such as sex, but we do not have enough data to validate these conjectures. Furthermore, publication bias was not obvious in our results.

## Conclusion

In conclusion, our meta-analysis showed that the 1-year OS rate of PG patients was lower than that of DG patients. However, the 3- and 5-year OS rates were different between eastern and western countries: the rates were lower in PG patients than in DG patients in eastern countries, but there was no significant difference in western countries. This result can provide evidence for the prognostic evaluation of GC after gastrectomy.

## Data Availability

All studies were retrieved from PubMed, EMBASE, Web of Science, Cochrane Library, China National Knowledge Infrastructure (CNKI), Wanfang, and VIP databases.

## Abbreviations

OS: Overall survival PG Proximal gastric cancer DG Distal gastric cancer GC Gastric cancer NOS Newcastle-Ottawa Scale RR Relative risk
CI: Confidence interval *I*^2^ *I* square statistic
